# Window-Based Energy Selecting X-ray Imaging and Charge Sharing in Cadmium Zinc Telluride Linear Array Detectors for Contaminant Detection

**DOI:** 10.3390/s23063196

**Published:** 2023-03-16

**Authors:** Antonino Buttacavoli, Fabio Principato, Gaetano Gerardi, Donato Cascio, Giuseppe Raso, Manuele Bettelli, Andrea Zappettini, Vincenzo Taormina, Leonardo Abbene

**Affiliations:** 1 Department of Physics and Chemistry (DiFC)—Emilio Segrè, University of Palermo, 90128 Palermo, Italy; antonino.buttacavoli@unipa.it (A.B.);; 2 IMEM/CNR, 43100 Parma, Italy; 3 Department of Mathematics and Informatics, University of Palermo, 90123 Palermo, Italy

**Keywords:** CZT detectors, charge sharing, semiconductor pixel detectors, X-ray detectors, energy-resolved X-ray imaging, contaminant detection

## Abstract

The spectroscopic and imaging performance of energy-resolved photon counting detectors, based on new sub-millimetre boron oxide encapsulated vertical Bridgman cadmium zinc telluride linear arrays, are presented in this work. The activities are in the framework of the AVATAR X project, planning the development of X-ray scanners for contaminant detection in food industry. The detectors, characterized by high spatial (250 µm) and energy (<3 keV) resolution, allow spectral X-ray imaging with interesting image quality improvements. The effects of charge sharing and energy-resolved techniques on contrast-to-noise ratio (CNR) enhancements are investigated. The benefits of a new energy-resolved X-ray imaging approach, termed window-based energy selecting, in the detection of low- and high-density contaminants are also shown.

## 1. Introduction

The importance of energy-resolved photon counting (ERPC) systems for quality enhancements in X-ray images is now widely recognized [1,2,3,4,5,6,7,8]. Due to the energy-dependence of the X-ray attenuation processes, spectral X-ray imaging represents a key tool for high resolution material detection and quantitative analysis, especially for medical diagnosis [4,5,9,10] and non-destructive testing (NDT) in security and food industry [6,7,8]. Once the spectral images are acquired, different techniques and algorithms can be easily applied to combine and weight the energy-binned images. While the advantages of the energy-resolved approach have been well demonstrated in several studies [9,10,11,12], the development of ERPC systems with high spatial and energy resolution is still currently under research and development. ERPC prototypes based on room temperature compound semiconductor detectors have given the best results, ensuring direct photon counting detection and good room temperature energy/spatial resolution [1,2,3,4,5,6,7,8]. Among these materials, cadmium zinc telluride (CdZnTe or CZT) has reached an excellent maturity level in room temperature X-ray and gamma-ray detection from photon energies of few keV up to 1 MeV [1,2,3,4,5,6,7,8]. Recently, in the framework of the *AVATAR X project* (funded by the Italian Ministry for University and Research), we developed ERPC systems based on sub-millimetre CZT linear array detectors for contaminant detection in food industry. In this work, we will present the main results obtained from the developed ERPC prototypes, in terms of both detector and X-ray imaging performance. The first part focused on the characterization of the spectroscopic performance of the new CZT detectors, with particular attention to the charge sharing effects. In the second part, the results from contrast-to-noise ratio (*CNR*) enhancements in X-ray images will be shown. A new energy-resolved X-ray imaging approach, termed *window-based energy selecting*, was developed and comparisons with other energy weighting approaches will be presented. The results will show important improvements in low/high density contaminant detection, due to the reduced charge sharing effects from the linear array layout together with the new energy-resolved X-ray imaging technique.

## 2. The CZT-Based ERPC Prototype

### 2.1. The CZT Linear Array Detector

**Figure 1:**
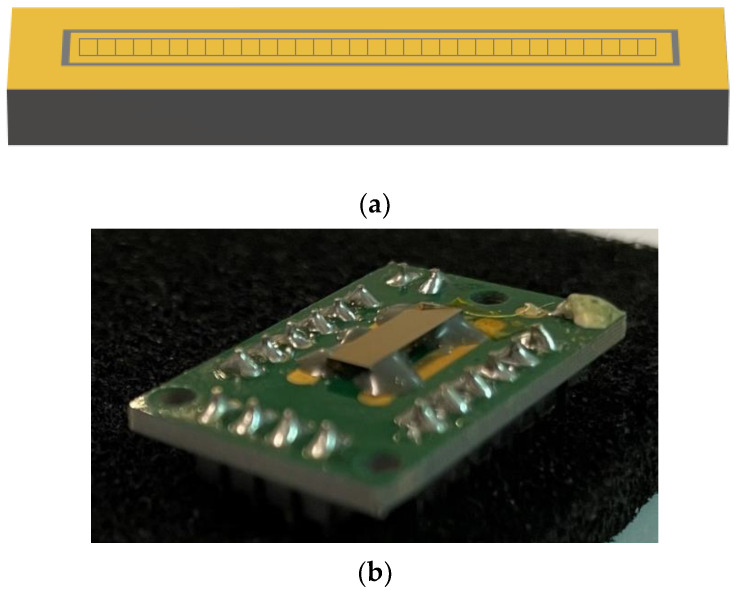
(**a**) The anode layout of the CZT linear array detector (3.0 × 10.4 × 1.1 mm^3^). The linear array is characterized by 32 pixels (225 µm) with a pitch of 250 µm. The width of the inter-pixel gaps is equal to 25 µm for all pixels. The pixel array is surrounded by internal and external guard-rings. (**b**) A picture of the detector from the cathode side.

A CZT linear array was fabricated by IMEM-CNR of Parma (Italy, http://www.imem.cnr.it, accessed on 14 March 2023) and due2lab s.r.l. (Reggio Emilia, Italy; http://www.due2lab.com, accessed on 14 March 2023). The detector was based on a CZT crystal (3.0 × 10.4 × 1.1 mm^3^) grown by the boron oxide encapsulated vertical Bridgman (B-VB) growth technique [13,14,15,16,17,18]. Recently, B-VB CZT detectors with pixel and planar electrode geometries have been successfully realized at IMEM-CNR of Parma, Italy [13,14,15,16,17,18]. Generally, the detectors, equipped with quasi-ohmic gold electroless contacts, are characterized by very low leakage current (<100 pA at high bias voltage of 1000 V) and no bias-induced polarization effects [19,20,21,22]. Interesting room temperature energy resolutions were obtained, ranging from 0.6 keV [18] to 2.4 keV [15] FWHM at 59.5 keV, depending on the noise-characteristics of the front-end electronics used. The good charge transport properties (mobility-lifetime products of electrons µτ_e_ > 10^−3^ cm^2^/V) beside the high-bias voltage operation (>7000 V/cm) allowed the minimization of the effects of high-flux radiation induced polarization in the detectors, with no spectral degradations up to 1 Mcps at 60 keV [15]. Figure 1 shows the anode layout of the detector which consisted in a linear array of 32 pixels with a pixel pitch of 250 µm, surrounded by two guard-rings (external and internal guard-rings), while the cathode side was a simple planar electrode. 

**Figure 2:**
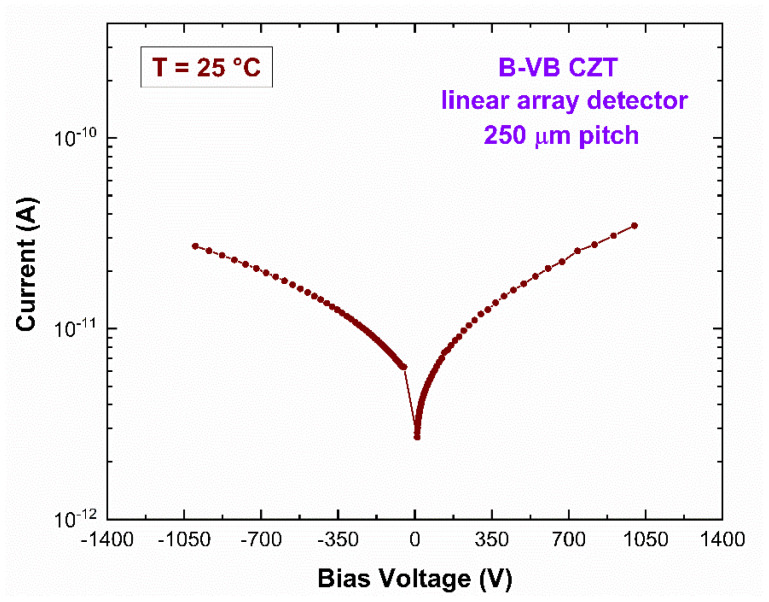
The current-voltage characteristics of a tested pixel of the CZT linear array detector. Low leakage current < 30 pA at −1000 V (9000 V/cm) was measured; the symmetry of the curve confirms the quasi-ohmic nature of the gold-electroless contacts.

To minimize the effects of charge sharing between the pixels, great efforts were made to reduce the inter-pixel gaps (<50 µm). In our case, an inter-pixel gap of 25 µm was realized. The external guard-ring was designed to minimize the surface leakage currents, while the internal guard-ring was realized to reduce the effects of charge sharing on the pixels. The internal guard-ring was coupled to the read-out electronics for coincidence measurements with the pixels. The rejection of the pixel events in temporal coincidence with the internal guard-ring emulated the effects of a slit collimator on the detector. The presence of a slit collimator should allow only the irradiation of the linear array, giving lower charge sharing than the pixel layout one. With this set-up, each pixel of the linear array should be influenced by the charge sharing from the 2 adjacent pixels, while for a standard pixel detector by the 8 neighbouring pixels. Concerning the leakage current of the pixels, very low leakage current values (<30 pA at −1000 V) were measured, as reported in Figure 2.

### 2.2. The Readout Electronics

**Figure 3:**
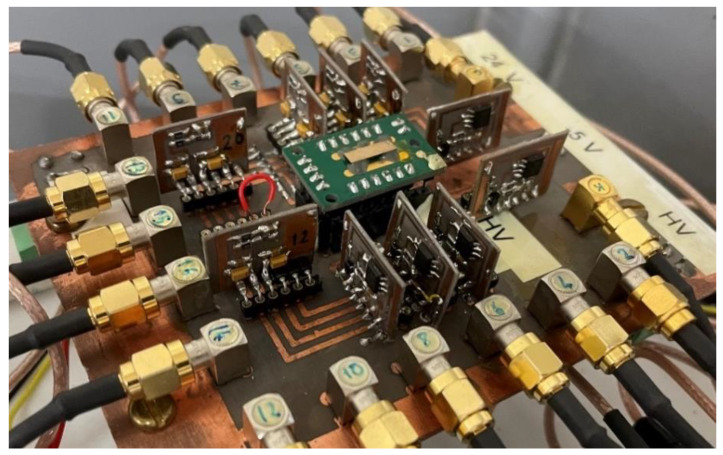
The B-VB CZT linear array detector coupled to the charge sensitive preamplifiers (CSPs).

The pixels (12 pixels), the internal guard-ring and the cathode electrodes were coupled to hybrid charge-sensitive preamplifiers (CSPs) and processed by a 16-channel digital pulse processing (DPP) electronics. Both the CSPs and the digital electronics were developed at DiFC of the University of Palermo (Italy). The CSPs were characterized by an equivalent noise charge (ENC) of about 100 electrons and equipped with a resistive-feedback circuit with a decay time constant of 20 µs. The pixels and the internal guard-ring were DC coupled to the CSPs, while the cathode was AC-coupled. Figure 3 shows the board with the CZT detector (cathode electrode view) and the hybrid CSPs. The digital electronics consisted in four digitizers (DT5724, 16 bit, 100 MS/s, CAEN S.p.A., Italy http://www.caen.it, accessed on 14 March 2023) driven by an original firmware developed by our group [23,24,25]. The digital pulse processing was based on a fast shaping (single delay line), for pulse timing and counting analysis, and a slow processing (trapezoidal shaping) optimized for pulse height and shape analysis.

## 3. The Spectroscopic Response of the CZT-Based ERPC Prototype

**Figure 4:**
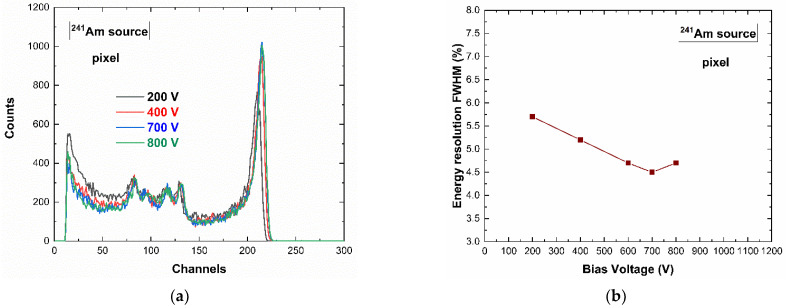
(**a**) ^241^Am energy spectra of a tested pixel at different bias voltages; the low-energy background, due to the charge sharing effects, is reduced by increasing the voltage. (**b**) The energy resolution FWHM at 59.5 keV vs. the bias voltage: the best energy resolution is obtained at −700 V.

The spectroscopic performance of the system was investigated at room temperature (T = 20 °C) by using uncollimated radiation sources (^109^Cd, ^241^Am, ^57^Co). Figure 4 shows the measured energy spectra (^241^Am source) for a tested pixel (pixel no. 6) at different cathode bias voltages (negative bias voltage). The best energy resolution, equal to 4.5% (2.7 keV) FWHM at 59.5 keV, was obtained at −700 V.

**Figure 5:**
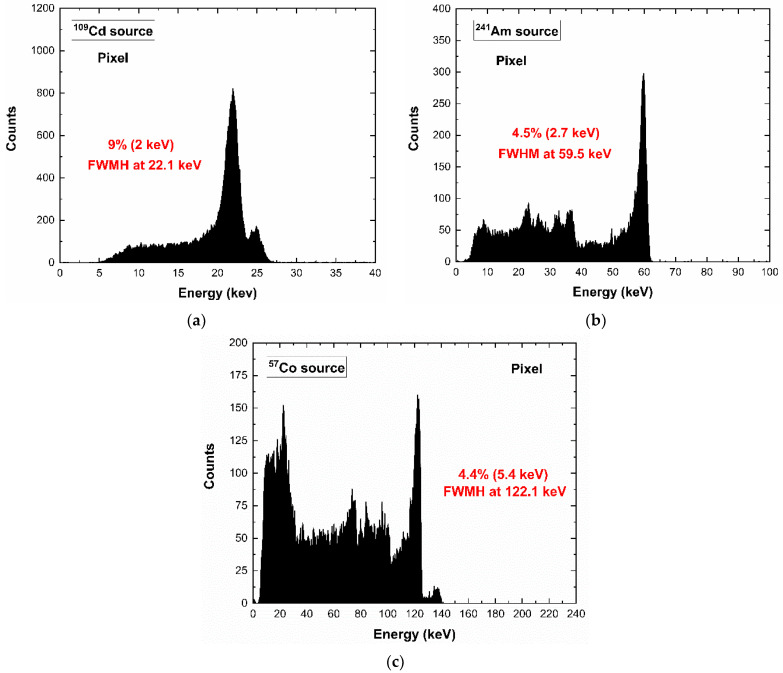
(**a**) ^109^Cd, (**b**) ^241^Am, (**c**) ^57^Co energy spectra of a tested pixel at room temperature; the low-energy background and the peaks at 23.2 and 27.5 keV are due to the charge sharing effects and fluorescence crosstalk.

Figure 5 shows the energy spectra under different radiation sources: the energy lines of 22.1 and 24.9 keV of the ^109^Cd source, the 59.5 keV line of ^241^Am source and 122.1 and 136.5 keV of the ^57^Co source. The background at low energies was due to the charge sharing, while the energy peaks at 23.2 and 27.5 keV represent the fluorescent X rays (Cd-Kα and Te- Kα X-rays) from the adjacent pixels and internal-guard-ring.

**Figure 6:**
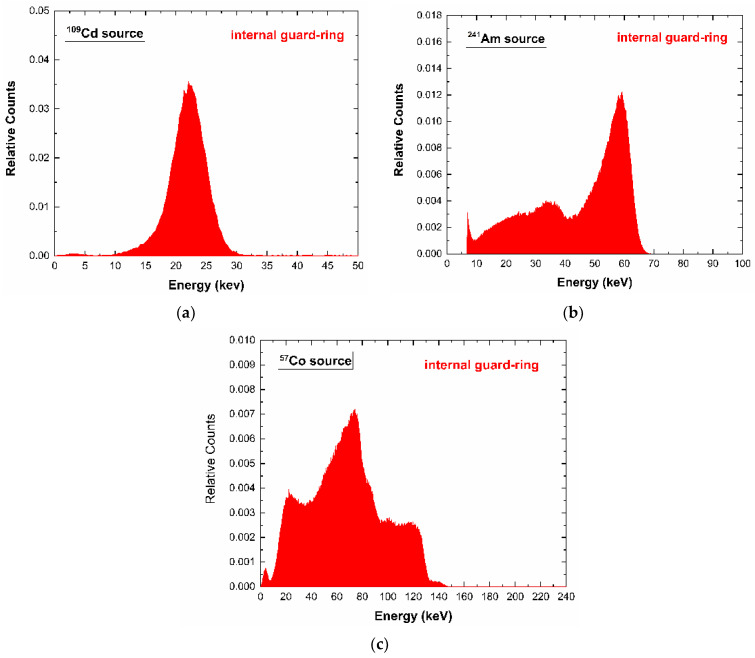
(**a**) ^109^Cd, (**b**) ^241^Am, (**c**) ^57^Co energy spectra of the internals guard-ring.

**Figure 7:**
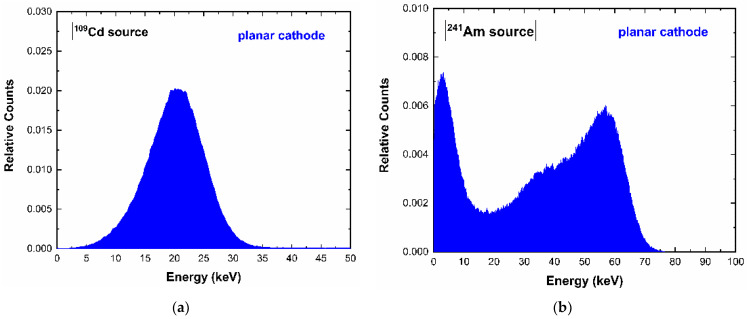

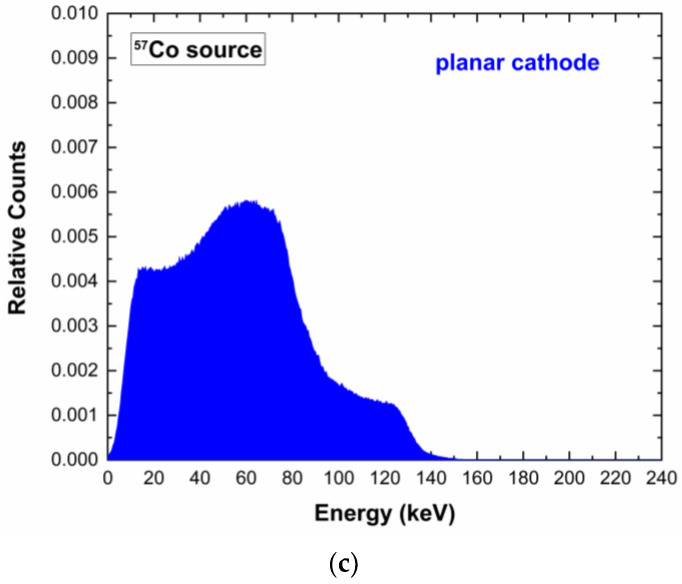
(**a**) ^109^Cd, (**b**) ^241^Am, (**c**) ^57^Co energy spectra of the planar cathode electrode.

The system was also able to provide the energy spectra from the internal guard-ring and the planar cathode (Figure 6 and Figure 7). The energy resolution was degraded, if compared to the pixel one, mainly due to the increased leakage current/capacitance and the reduction in the benefits in charge collection from the small pixel effect [5]. As will be discussed in the following section, the signals from the internal guard-ring and the planar cathode can be very helpful to compensate the spectral and counting distortions from charge sharing and incomplete charge collection. 

## 4. Charge Sharing and Incomplete Charge Collection Effects 

### 4.1. Charge Sharing Measurements 

**Figure 8:**
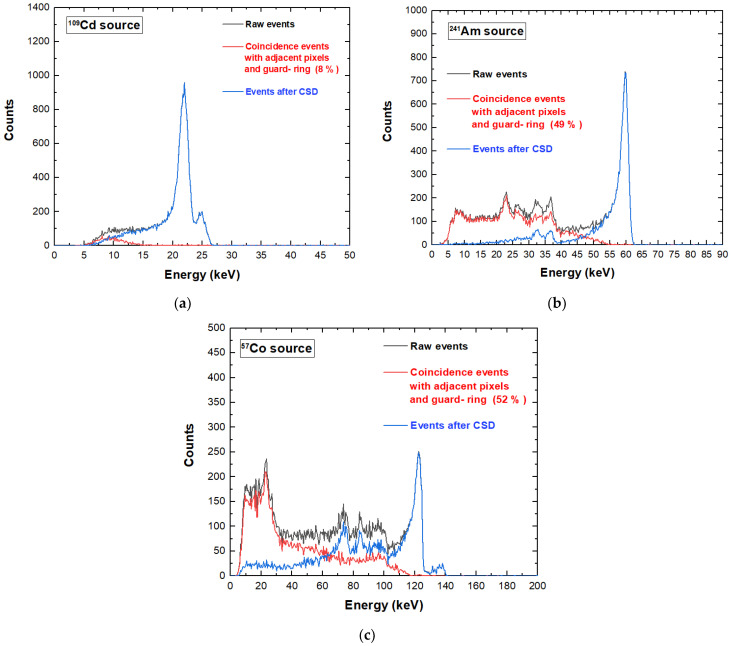
Charge sharing measurements of a tested pixel under (**a**) ^109^Cd, (**b**) ^241^Am and (**c**) ^57^Co sources. The blue lines represent the uncollimated energy spectra after charge sharing discrimination (CSD). The raw spectra (black line) of all events and the spectra of the coincidence events with the two adjacent pixels and the internal guard-ring (red line) are also shown.

Charge sharing investigations were performed by detecting the events of each pixel in temporal coincidence with the two adjacent pixels and the internal guard-ring. The coincidence events were measured within a coincidence time window (CTW) of 300 ns, ensuring the full detection of all events. The charge sharing results for a tested pixel under ^109^Cd, ^241^Am, and ^57^Co sources are shown in Figure 8. Three energy spectra are presented: the raw spectrum containing all events (black line), the spectrum of the coincidence events of the pixel with the two adjacent pixels and the internal guard-ring (red line) and the spectrum of the single events (i.e., the events with multiplicity *m* = 1). The single-event spectrum (blue line) was obtained after the rejection of the coincidence events, i.e., after the application of the charge sharing discrimination (CSD) technique [17,23]. As is well known, this technique simply consists in rejecting, from the energy spectrum of a selected pixel, the events that are in temporal coincidence with the neighbouring pixels (in our case with the two adjacent pixels and the internal guard-ring). Concerning the energy spectra of Figure 8, the spectra after CSD (blue lines) were obtained by subtracting the red spectra from the black ones. The shape of the energy spectrum was strongly improved after CSD. The coincidence events (red line) were mainly due to charge sharing and fluorescence crosstalk effects: the fluorescent peaks were at 23.2 and 27.5 keV, the escape peaks were at 36.3, and 32 keV (^241^Am), the low-energy background and tailing. At energies greater than the K-shell absorption energy of the CZT material (26.7 keV, 9.7 keV and 31.8 keV for Cd, Zn and Te, respectively), the presence of fluorescent X rays created an increase in the coincidence percentages.

**Figure 9:**
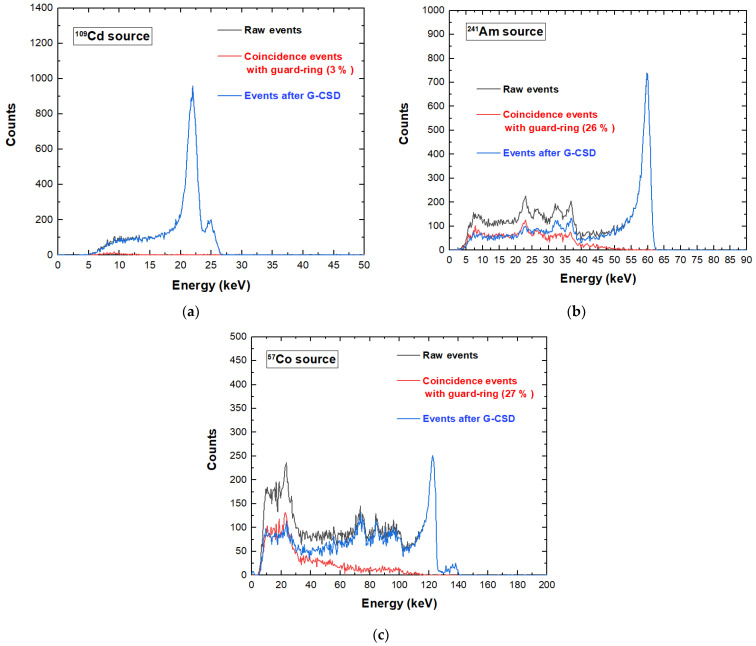
Charge sharing measurements of a tested pixel under (**a**) ^109^Cd, (**b**) ^241^Am and (**c**) ^57^Co sources. The blue lines represent the uncollimated energy spectra after charge sharing discrimination with the internal-guard ring (G-CSD). The raw spectra (black line) of all events and the spectra of the coincidence events with the internal guard-ring (red line) are also shown.

**Figure 10:**
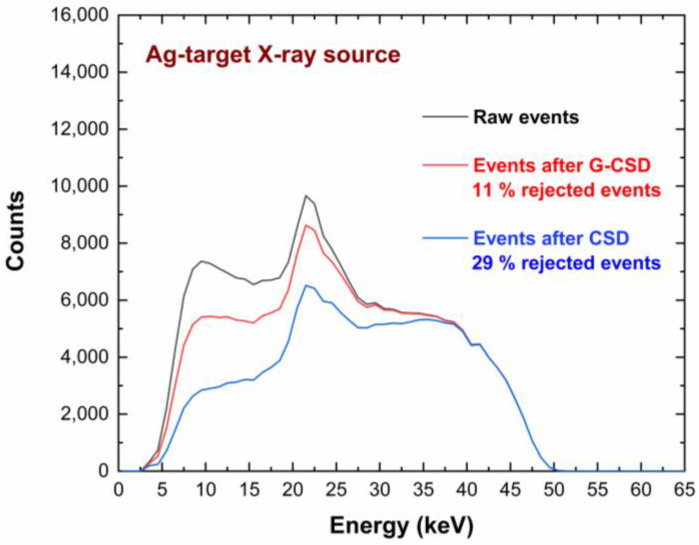
Charge sharing measurements of a tested pixel under Ag-target X-ray tube source. The red and blue lines represent the uncollimated energy spectra after G-CSD and CSD, respectively. The raw spectrum (black line) is also shown.

A key result concerns the global reduction of the coincidence/charge sharing percentages, if compared with similar CZT pixel arrays with wider inter-pixel gap of 50 µm (charge sharing percentages > 70% at 60 keV) [23]. We obtained coincidence percentages of 8%, 49%, and 52% under ^109^Cd, ^241^Am and ^57^Co, respectively. This reduction was due to smaller ratio between the inter-pixel gap area and the pixel area. As discussed before, we can also read the signals from the internal guard-ring and evaluate the charge sharing with the pixels. Figure 9 shows the effects of rejecting only the coincidence events with the internal guard-ring (i.e., after G-CSD). In this case, the coincidence detection with the internal guard-ring could be used to emulate the effects of the presence of a slit collimator, allowing only the charge sharing events between adjacent pixels. This represents the desired working set-up for the linear X-ray scanner, where the only charge sharing events between adjacent pixels will influence the acquired images. Charge sharing effects were also investigated on poly-energetic X-ray spectra. We measured X-ray spectra from an X-ray tube (Mini-X, Amptek, Bedford, MA, USA) with a silver (Ag) target and a focal spot size of 2 mm, working at a voltage of 50 kV and tube current of 5 µA. The shape of the X-ray spectrum was also influenced by the 100 µm thick Al window of the detector. The results, after G-CSD and CSD, are shown in Figure 10. It is clearly visible that charge sharing strongly influenced the low-energy region of the X-ray spectra. The effects of these different charge sharing conditions on the image quality will be analysed and discussed in Section 5.

### 4.2. Incomplete Charge Collection in Single Events 

**Figure 11:**
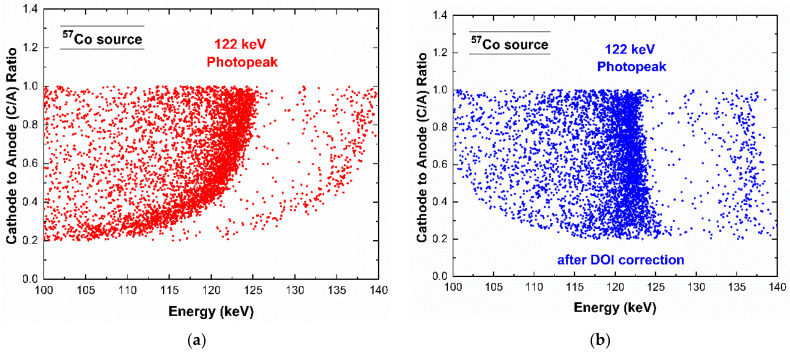

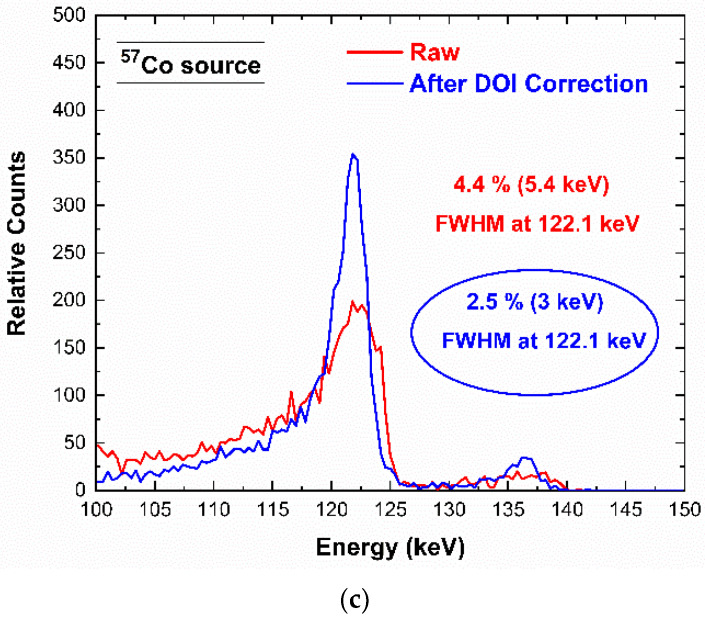
(**a**) Scatter plot of the cathode to anode (C/A) ratio vs. the energy of the anode (pixel). (**b**) The scatter plot after depth of interaction (DOI) correction. (**c**) The raw (red line) and corrected (blue line) energy spectra of the events of the scatter plots. Interesting energy resolution improvements are obtained after the DOI correction.

As shown in Figure 8 and Figure 9, a tailing in the main energy peaks was clearly visible, and more pronounced at high energies. The tailing, due to incomplete charge collection effects, was more severe in signals with a high hole contribute (hole trapping); this occurred for photon interactions near the anode (pixel), due to the shape of the weighting potential of the pixels [23]. We performed a correction of these incomplete charge collection effects by exploiting the coincidence events with the cathode electrode. We used the traditional cathode to anode ratio (C/A) [26,27,28,29] and its relationship with the energy of the photons from the pixels (anodes), as shown in Figure 11 for the ^57^Co events. The analysis was applied to single events (*m* = 1). The curvature of Figure 11a clearly highlights that photons interacting near the pixel, i.e., characterized by low C/A values, presented more incomplete charge collection effects. By modelling the curve of Figure 11a [26], it was possible to recovery the energy as shown in Figure 11b,c. This technique is also called depth of interaction (DOI) correction. Improvements in the energy resolution are clearly visible.

### 4.3. Incomplete Charge Collection in Charge Sharing Events 

**Figure 12:**
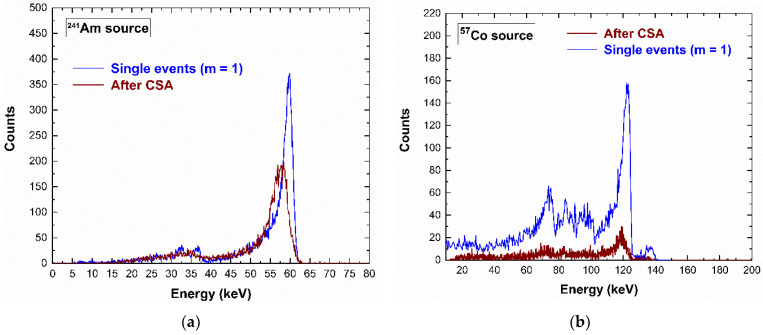
Measured (**a**) ^241^Am and (**b**) ^57^Co energy spectra after the application of the charge sharing addition (CSA) technique. The energy spectra of the single events (blue lines) and the spectra of the coincidence events with the two adjacent pixels (multiplicity *m* = 2) after CSA (brown lines). The energy spectra after CSA are characterized by energy deficits (2 keV at 59.5 keV and 3 keV at 122.1 keV), due to the presence of charge losses near the inter-pixel gaps.

**Figure 13:**
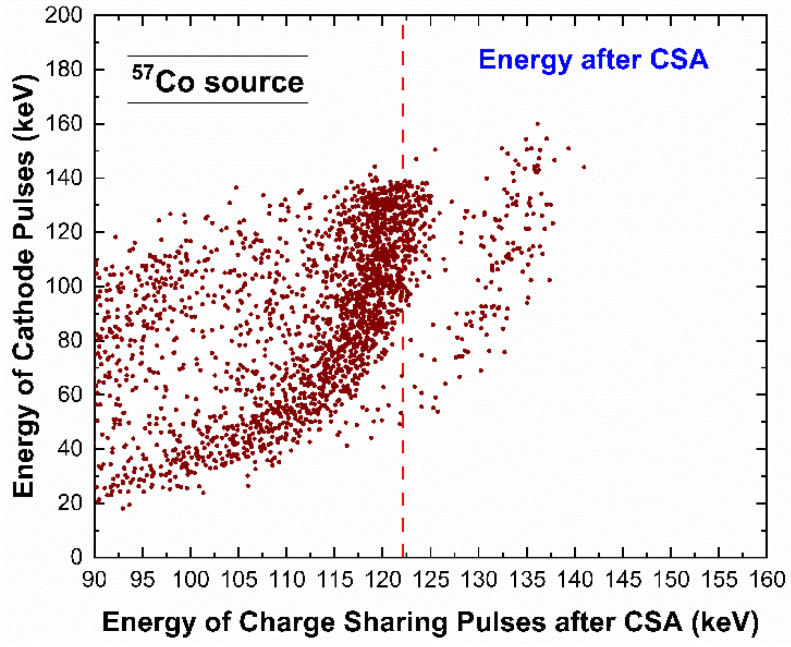
Scatter plot of the energy of the cathode events vs. the energy of the shared events after CSA. The presence of energy deficits (about 2 keV at 122 keV), even for event interacting near the cathode (i.e., characterized by the full cathode energy of 122 keV and very low hole contribute to the signal), clearly demonstrates that these losses are not related to the hole trapping, but to electric field line distortions at the inter-pixel gap. The dashed red line represents the correct energy after CSA (i.e., 122.1 keV).

As is well documented in the literature [30,31,32,33,34], the charge sharing events, rejected by CSD, can be recovered in counting and energy through the application of the charge sharing addition (CSA) technique. This technique consists in a simple addition of the energy of the charge sharing events. Unfortunately, the summed energy is often characterized by deficits due to incomplete charge collection effects at the inter-pixel gap [30,31,32,33,34]. These energy deficits were also observed in our measurements, as shown in Figure 12. The energy spectra after CSA were characterized by energy deficits of 2 keV at 59.5 keV and 3 keV at 122.1 keV. Generally, the deficit was lower than that of detectors with 50 µm inter-pixel gap (5 keV at 59.5 keV), demonstrating the benefits in reducing this gap (25 µm inter-pixel gap in our detector). Concerning the origin of these charge losses, several interpretations have been given; some researchers attributed these effects to the hole trapping [33], others to the presence of distortions of the electric field lines near the inter-pixel gap [30,31,34]. To better clarify this issue, we measured the shared events after CSA in temporal coincidence with the cathode signals. Figure 13 shows the energy of the cathode pulses vs. the energy of the charge sharing events after CSA.

The curvature of the scatter plot, related to the 122 keV photopeak, highlights that the summed energy changes with the interaction depth (represented by the energy of the cathode). In particular, an energy deficit of about 2 keV at 122 keV was present even for photon interactions near the cathode, i.e., characterized by a full cathode energy of 122 keV. Because photon interactions near the cathode gave pulses with a small hole contribute, the hole trapping cannot justify the charge losses. This confirms that the presence of the energy deficit could be only due to distortions of the electric field lines near the inter-pixel gap.

## 5. Energy-Resolved Images and Contrast Enhancements in a Food Sample

In this section we will present the results from different energy-resolved approaches on the image quality enhancements. The effects of charge sharing in both image segmentation and quality will be also shown. The images from a simple example of a food product were acquired and analyzed.

### 5.1. Experimental Set-Up and Data Acquisition

**Figure 14:**
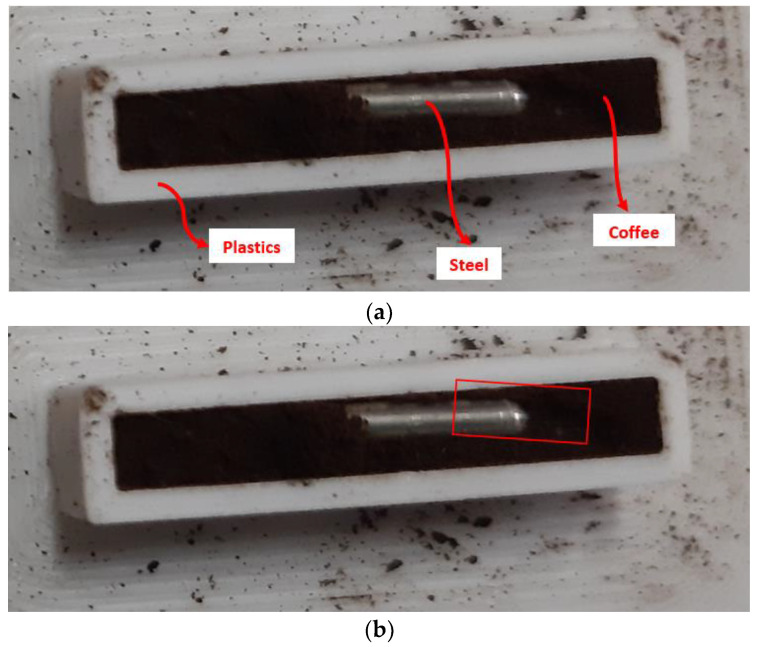
(**a**) Visible image of the phantom used for X-ray imaging measurements: a plastic container with coffee powder and a steel sample. (**b**) The red rectangular line highlights the scanning area.

X-ray images were acquired from the phantom of Figure 14 under the Ag-target X rays (50 kV), representing an example of a simple food product. We used a plastic (ABS: acrylonitrile butadiene styrene) container (22 × 5 × 5 mm^3^) with coffee powder and the presence of a steel pseudo-cylinder (*ϕ* = 1 mm; 10 mm length), as example of high-density contaminant. The plastic edge of the container was also imaged and used as example of low-density contaminant. The scanning area (2.5 × 7.75 mm^2^) is highlighted in Figure 14b by the red rectangular line. The phantom was positioned at a distance of 7 cm from both the detector and the X-ray tube. Each X-ray image, constituted by 310 pixels, was obtained from 31 acquisitions of the 10 pixels of the linear array. Each acquisition, performed at different positions with steps of 225 µm, was done in stationary conditions, i.e., by stopping the system (X-ray tube and detector) at each acquisition. Each image was characterized by three key materials: the coffee powder considered as background and the steel/plastics as contaminants. To quantify the effects of charge sharing on the image quality, we analysed three different image types, characterized by different presence-levels of charge sharing:

1. images with the presence of charge sharing in each pixel (*Raw images*);
2. images including only the charge sharing between adjacent pixels and rejecting the charge sharing with the internal guard-ring (*G-CSD images*);
3. images after full charge sharing discrimination (CSD), i.e., after rejecting all charge sharing events in each pixel (*CSD images*).

### 5.2. Image Processing and Figure of Merit for Image Quality

**Figure 15:**
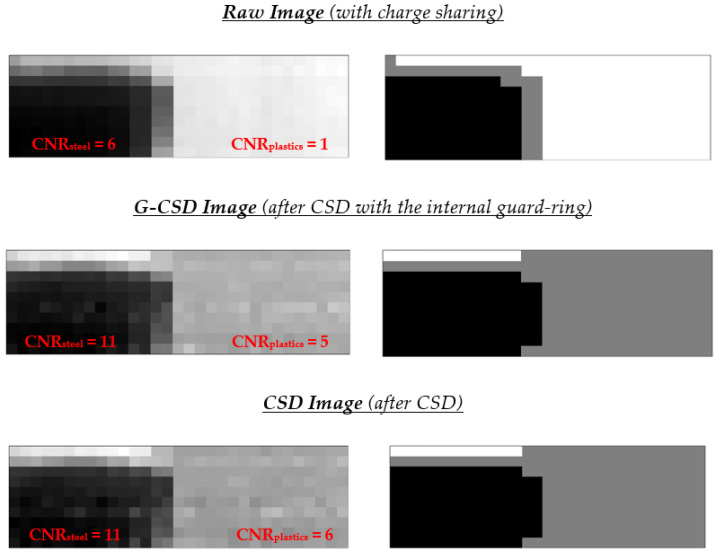
On the left side, the grayscale raw, the G-CSD and the CSD images from the tested phantom, presented in photon counting (PC) mode. On the right side, the results of the material segmentation (k-means clustering): the gray zone for coffee, white for plastics and black for steel. The segmentation is well done after partial (G-CSD) and total rejection (CSD) of the charge sharing events, while a poor segmentation is obtained with the raw image (on the first row of the right-side column). The *CNR* values are also reported.

A key processing step concerned the material segmentation of the images, i.e., the selection of the regions of interest (ROIs) for each investigated material (coffee, steel and plastics). This was performed by using an unsupervised learning method, implemented using the k-means++ algorithm [35,36,37,38]. The k-means algorithm is an unsupervised machine learning approach, capable of working without training data. By selecting the number of clusters *k*, the method assigned each data point to the *k-th* cluster minimizing the distance metric between k-centroids. Compared to k-means, the k-means++ algorithm allows the choice of initial seeds in order to make the clustering process more robust. In our case, we applied the squared Euclidean distance metric for *k =* 3 (coffee, steel, plastics) and a very simple randomized seeding technique was used [38]. Figure 15 shows the three image types (Raw, G-CSD and CSD images) and the results after material segmentation. The grayscale images on the left side of Figure 15 were obtained through the photon counting (PC) mode, i.e., by using for each pixel the total counts over the entire energy spectrum (5–50 keV), normalized to the total counts without the phantom. On the right side, the material segmentation (black area for steel, gray for coffee and white for plastics) performed on low charge sharing images (G-CSD and CSD images) was in agreement with the ground-truth segmentation; meanwhile, poor segmentation was obtained in the raw images. To quantify the image quality enhancements, we used the well-known contrast-to-noise ratio (*CNR*) [6,7,8] as figure of merit, calculated for each ROI selected by the segmentation, as follows:

(1) $$CNR_{C}=\frac{\left|I_{C}-I_{B}\;\right|}{\sigma_{B}}=\frac{C_{c}}{\sigma_{B}}$$

where, *I_C_* and *I_B_* are the average normalized intensities of the selected contaminant (steel or plastics) and background (coffee), respectively, while *σ_B_* is the standard deviation of the background and *C_C_* the contrast. The *CNR* gave a good quantification of the contributes of both contrast and noise in the images. Typically, a *CNR* ranging between 3–5 is required for an object to be considered detectable [6,7,8].

The *CNR_plastics_* values were mainly influenced by charge sharing, the effects of which are prevalent at low energies; after CSD, the *CNR_plastics_* increased by 500% and by 80% for steel. Moreover, even the image with moderate charge sharing (G-CSD image) allowed good *CNR* enhancements for both contaminants. 

### 5.3. Energy-Resolved Imaging and Contrast Enhancements

Once a photon counting (PC) image was acquired, the system was able to split the PC image in several energy-binned images. In our case, each PC image was composed by *N* = 45 energy-bin images (binned at 1 keV), covering the full energy spectrum (5–50 keV). In this section, we will present further *CNR* improvements after the application of different energy-resolved approaches. A class of energy-resolved methods is represented by the *energy weighting approaches* [6,7,8,9], which consisted in assigning weights *w* to the different energy bins that compose the measured spectrum, before generating a spectrum-integrated final image. The final image was a linear combination of the energy-bin images (Equation (2)), with the weights *w(E)* typically chosen to enhance the *CNR*. Among these, the *image-based energy weighting* [39] allowed interesting *CNR* improvements, especially when compared with the *projection-based energy weighting* [40]. In the projection-based energy weighting the weights *w* ∝ *E*^−3^ followed the behaviour of the linear attenuation coefficients with the energy; this method over-emphasized the low-energy region of the spectrum, while de-emphasizing the high-energy region significantly. This approach requires particular attention on the selection of the low energy threshold; in fact, the similar counting intensities at very low energies (<10 keV), even for different materials, can often create poor *CNR* in the images. As already demonstrated in the literature [39], the optimal weight for the image-based energy weighting approach is proportional to the contrast-to-noise-variance ratio (*CNVR*), which emphasises the energy-bins with low noise and high contrast *C*. The following equations describe the details of the two energy weighting approaches:

(2) $$Image_{weighted}=\overset{N}{\underset{i=1}{\sum }}w(E_{i})\cdot Image(E_{i})$$

(3) $$\;w(E_{i})=\frac{E_{i}^{-3}}{\sum_{j=1}^{N}E_{j}^{-3}}\;\;\;\;\;\;\;\;\;\mathrm{for}\mathrm{the}\mathrm{projection-based}\;\mathrm{energy}\;\mathrm{weighting}$$

(4) $$w(E_{i})=\frac{C(E_{i})\cdot \;\sigma_{i}^{-2}}{\sum_{j=1}^{N}C_{j}\;\cdot \;\sigma_{j}^{-2}}=\frac{CNVR\;\left(E_{i}\right)}{\sum_{j=1}^{N}CNVR\;\left(E_{j}\right)}\;\;\;\;\;\;\;\mathrm{for}\mathrm{the}\mathrm{image-based}\;\mathrm{energy}\;\mathrm{weighting}$$

Beside these two techniques, we also proposed a new method, termed *window-based energy selecting*, consisting of the selection of dedicated energy windows for each material, properly selected to enhance the *CNR* values. 

**Figure 16:**
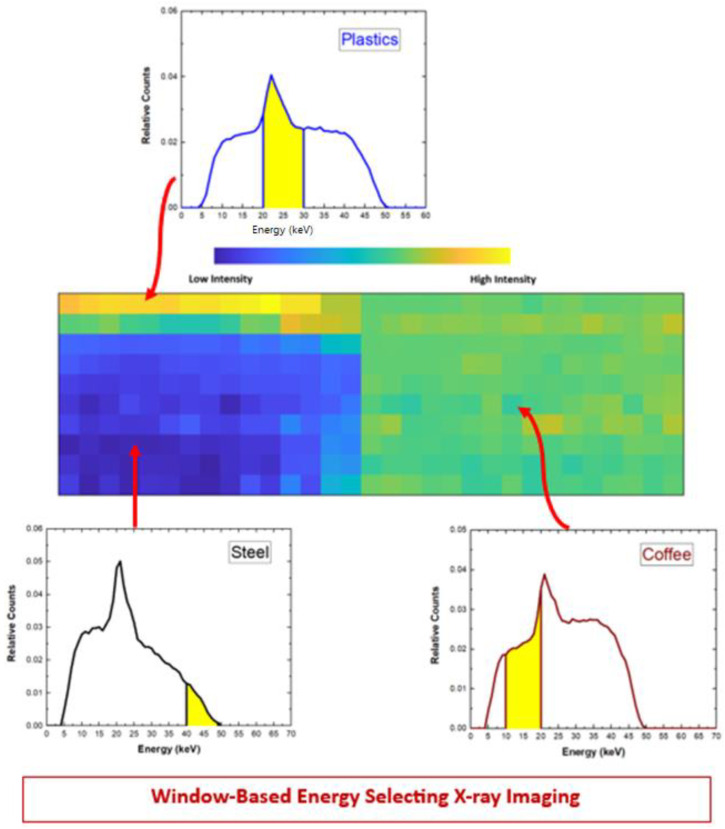
General overview of the *window-based energy selecting X-ray imaging approach*. The energy spectra of the pixels related to the three material zones (plastics, steel and coffee). The image is obtained by using the counts within the energy windows (yellow areas), selected to enhance the *CNR*. This technique, taking into account *CNR* enhancements between contaminants (steel and plastics) and background (coffee), foresees the selection of the optimal width and position of the energy windows.

The final image was created with the counts of the selected windows for each material (contaminants and background). Figure 16 shows a general overview of the *window-based energy selecting* technique used in this work. A similar approach was used in K-edge X-ray imaging by selecting appropriate energy windows around the K-edge jump [41] for *CNR* enhancements. In this case [41], the optimization concerned the selection of the optimal width for the energy windows at a fixed position in the energy spectrum, i.e., always positioned at the left and right sides of K-edge. Our technique was able to optimize both the width and the position of the energy windows, taking into account *CNR* enhancements between the contaminants (plastics and streel) and the background (coffee). The optimal energy windows were selected through an iterative approach, as follows:

- first, we fixed the same width *w* of the energy windows for both contaminant and background; in our case we were able to select 40 different energy-window widths, with a step of 1 keV, within the energy range *R* of 45 keV (5–50 keV); the minimum *w* was fixed to 5 keV, taking into account the energy resolution of the system (*w* ≥ 2·*FWHM*~5 keV within the 50–5 keV energy range); we stress that the use of a *w* of 45 keV, for both contaminant and background, was equivalent to the application of the photon counting (PC) mode;
- second, for each fixed *w* value, we selected the optimal position of the windows in the energy range, taking into account the highest *CNR* value among the values obtained from all possible positions (step of 1 keV); for example, by using two window widths *ws* of 44 keV, the number of possible position values was 4 (two different positions for the contaminant and two for the background), by using two *ws* of 43 keV, 9 positions and by using two generic *ws*, we could analyze *(R-w +* 1)^2^ different positions.

**Figure 17:**
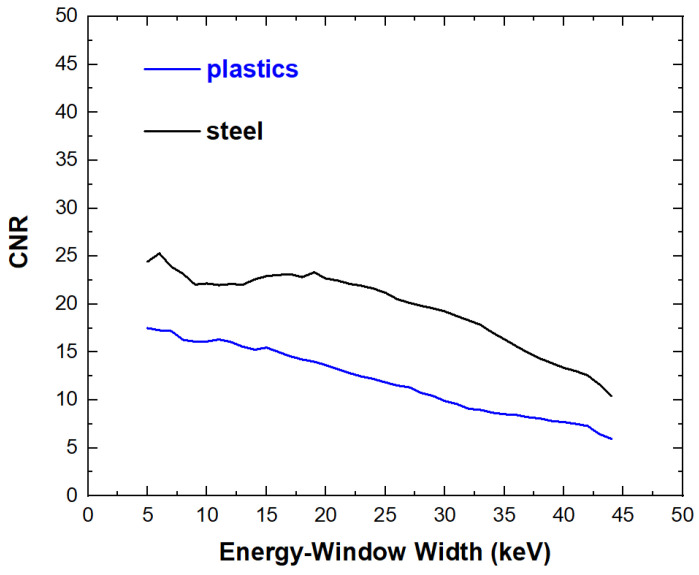
*CNR* values vs. the widths of the energy windows of the *window-based energy selecting approach*.

**Figure 18:**
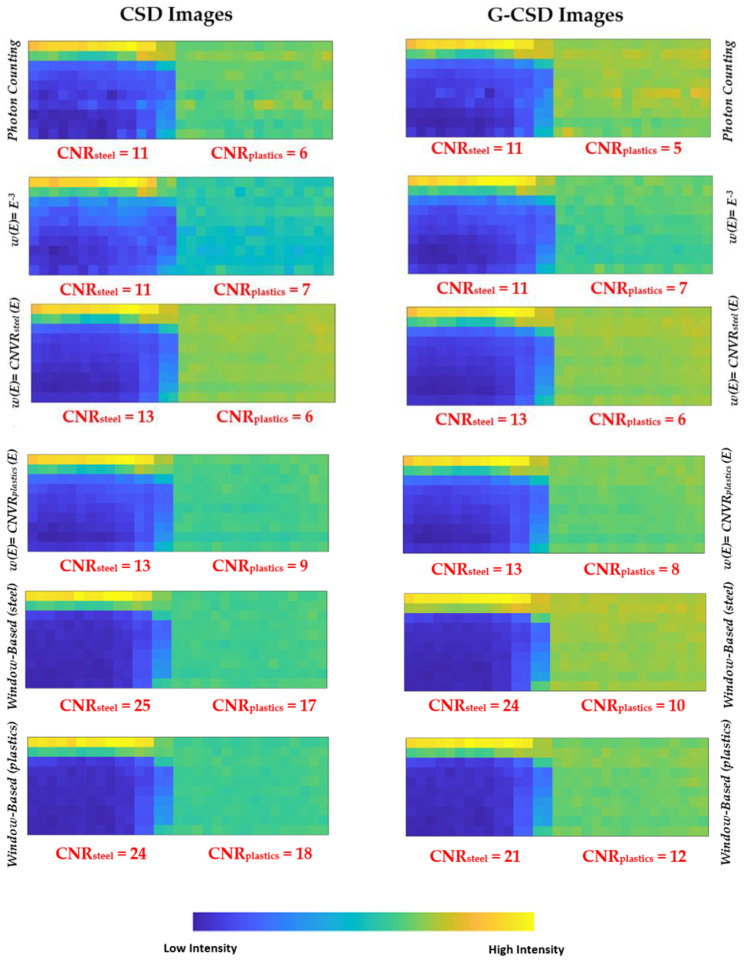
The images and the *CNR* values (steel and plastics contaminants on coffee) presented in photon counting (PC) mode and after the application of different energy-resolved approaches. The CSD and G-CSD images on the left and right sides, respectively. Generally, two images are obtained, one optimized for steel and the other for plastics detection.

Concerning the selection of the energy-window widths, we observed that the best *CNR* values were obtained by using window widths as small as possible. *CNR* values improved quasi-monotonically by reducing the widths of the energy windows, as shown in Figure 17. In our case, the best *CNR* values were obtained by using energy windows of 5 keV. Despite narrow windows would result in fewer photons and high noise, these allowed higher *CNR* values. Figure 18 shows the results obtained by applying photon counting and energy-resolved approaches in CSD and G-CSD images. 

Generally, the energy-resolved approaches gave better *CNR* values than the photon counting one. Among these, low improvements were obtained from the projection-based energy weighting approach (*w* ∝ *E*^−3^), requiring energy threshold greater than 10 keV. For the other two energy-resolved approaches, two images were obtained, one optimized for steel and the other for plastics detection. 

The window-based energy selecting approach allowed the best results, with energy-windows of 5 keV. For example, the *window-based_plastics_* approach applied to the G-CSD images produced the best results with energy-windows of (45–50 keV), (25–30 keV), (18–23 keV), for steel, plastics and coffee, respectively. The effects of charge sharing between adjacent pixels (G-CSD images) on *CNR* values were quite moderate; charge sharing, with its high contribute at low energies, mainly influenced the *CNR_plastics_*, more sensitive to low energy photons.

## 6. Discussion and Conclusions

The spectroscopic and imaging performance of new sub-millimetre B-VB CZT linear array detectors, as ERPC prototypes for spectral X-ray imaging, were presented in this work. The activities are in the framework of *AVATAR X* project (funded by the Italian Ministry for University and Research), planning the development of X-ray scanners for contaminant detection in food industry. The key results from the investigations mainly concerned new knowledge about charge sharing effects in the CZT linear array detectors and interesting *CNR* enhancements in contaminant detection obtained with a new energy-resolved X-ray imaging approach. The results are summarized as follows:

- the realization of inter-pixel gap of 25 µm on pixel pitches of 250 µm allowed the reduction in charge sharing percentages (50% at 60 keV in our case; >70% at 60 keV with wider inter-pixel gaps) [23,30]; moreover, this also produced a reduction of the charge losses after CSA (2 keV at 60 keV in our case; >5 keV at 60 keV with wider inter-pixel gaps);
- the coincidence measurements of the shared events after CSA with the cathode events clearly highlighted that the charge losses after CSA are not related to the hole trapping, but they are due to distortions of the electric field lines near the inter-pixel gap;
- the good energy resolution (<3 keV) of the ERPC prototype allowed interesting image quality improvements after the application of energy-resolved techniques;
- a new energy-resolved approach, termed *window-based energy selecting*, was developed and excellent *CNR* enhancements in X-ray images were obtained, if compared with other energy-resolved techniques;
- the presence of charge sharing between adjacent pixels (G-CSD images) produced low distortions in *CNR*; *CNR_steel_* deteriorated of 4% and *CNR_plastics_* of 30% from images with no charge sharing (CSD images).

These investigations, despite performed at low photon counting rates (<1 kcps), gave useful knowledge even for high counting rate measurements (>1 Mcps), necessary to perform image acquisitions with low acquisition times as possible. In this work, we used low counting rates in our measurements to perform correct time coincidence analysis for charge sharing investigations. However, this allowed us to demonstrate that charge sharing between adjacent pixels produces moderate distortions in X-ray images and, therefore, the ERPC prototype (equipped with a slit collimator) can operate at high rates without charge sharing detection. Moreover, we foresee it working at high rates by coupling the detectors to new front-end electronics, recently developed by our group [18], allowing excellent energy resolution (<1 keV) even with very fast peaking times (<50 ns); this would allow us to work with very short-shaped pulses (*ballistic deficit pulse processing approach* [42]), ensuring excellent energy resolution and high throughput at high-rate measurements.

## Data Availability

Data is contained within the article.
